# COVID-19 affected the food behavior of different age groups in Chinese households

**DOI:** 10.1371/journal.pone.0260244

**Published:** 2021-12-17

**Authors:** Ting Chen, Chong Wang, Zhenling Cui, Xiaojie Liu, Jun Jiang, Jun Yin, Huajun Feng, Zhengxia Dou

**Affiliations:** 1 School of Environmental Science and Engineering, Zhejiang Gongshang University, Hangzhou, China; 2 Department of Clinical Studies, School of Veterinary Medicine, University of Pennsylvania, PA, United States of America; 3 Zhejiang Provincial Key Laboratory of Solid Waste Treatment and Recycling, Hangzhou, China; 4 Instrumental Analysis Center of Zhejiang Gongshang University, Hangzhou, China; 5 College of Resources and Environmental Sciences, China Agricultural University, Beijing, China; 6 Institute of Geographic Sciences and Natural Resource Research, Chinese Academy of Sciences, Beijing, China; 7 Hangzhou Urban Construction Investment Group Co., Ltd., Hangzhou, China; University of Florida, UNITED STATES

## Abstract

The COVID-19 pandemic brought profound changes to all corners of society and affected people in every aspect of their lives. This survey-based study investigated how household food related matters such as food sourcing and consumption behaviors of 2,126 Chinese consumers in different age groups changed approximately two months into the COVID-19 quarantine. A new food sourcing mechanism, community-based online group grocery-ordering (CoGGO), was widely adopted by households, particularly among the youngest group studied (18–24 years of age). The same group showed a higher confidence in the food supply system during the quarantine and a greater propensity for weight gain while staying-at-home. The more mature age group (≥35 years of age) showed heightened vigilance and awareness, with fewer grocery-shopping trips, a higher tendency for purchasing extra food, and less tendency to waste food. Survey findings of the new food-sourcing mechanism, attitudes to food, and changes in behavior among different age groups provide valuable insights to guide policies and management interventions to address matters pertaining to food supply and distribution, food access and household food security, and food waste reduction.

## Introduction

The COVID-19 pandemic brought profound changes to all corners of societies worldwide. For countless people complying with the stay-at-home orders and dealing with disrupted daily life, the ability to obtain food and keep themselves and their families properly fed was fundamental. Evidently, there was a phenomenal shift of household food-sourcing from in-store shopping to online-based purchases [1–5]. Online shopping was not new, but online shopping for food increased sharply due to limited physical access to stores or fear of virus exposure in public spaces [3, 4]. A Canadian study reported that only 1.5% of groceries were sold online before the COVID-19 pandemic, but that number grew to over 9.0% by the third week in March 2020 [5]. Additionally, Si et al. (2020) described grassroots efforts organized by citizens in Wuhan, the epicenter of the COVID-19 outbreak in China, to obtain food through buying groups during the strictly enforced lockdown [6].

Age has been known as an important socio-demographic factor pertaining to consumer food behavior [7], due to impacts related to economic endurance, convenience requirements, food hygiene attitude and health [8, 9]. For example, Ozen et al. (2012) reported that age influenced the purchasing and consumption behaviors for some foods, with older people showing a greater interest than younger ones in certain food type or items that are regarded as healthier [8]. Age has also been widely reported to have a strong impact on food waste behavior; older people tended to waste less food [9–13]. Although age has been found to be a modest predictor of food waste [14], the strength and direction of the relationship varies between different countries [15, 16]. The Findings of these pre-pandemic studies provided some insight into the impact of age on self-assessed food consumption and waste behaviors. However, systematic investigation of different age groups regarding household related food behaviors involving the whole spectrum spanning from food purchases to food usage and to waste is relatively scarce.

Household related food consumption behaviors involve a wide range of aspects, including food sourcing, food vigilance, food waste and the subsequent effects caused by food consumption, such as weight change etc. A number of recent studies shed some lights on addressing these issues. For example, there appeared to be a phenomenal shift that food sourcing changed dramatically with reduced physical trips to markets and increased online shopping because of the pandemic [1–6]. Several studies also examined household food behavioral change in terms of food choices and extra-quantity purchases, indicating elevated food vigilance. For instance, an Italian survey found that nearly half of the participants reported increased the consumption of comfort food, e.g. chocolate and dessert items [17]. A cross-national survey of 1,732 Chinese and 1,547 U.S. households reported purchasing extra amounts of various foods during the stay-at-home directives [2]. Moreover, the COVID-19 lockdown appeared to have induced a positive behavioral change regarding food wastage; 85% of 284 Tunisian respondents declared nothing of what they bought would be discarded, and most of the respondents have set up a strategy of saving, storing and eating leftovers [18]. Meanwhile, health-related implications such as weight change can be further exacerbated by overeating and reduced physical activities due to the home quarantine [19]. These studies collectively revealed the impact of pandemic isolation on various household related food behaviors, however consequences with different age groups remain unexplored.

The present study reports the findings of an online-based cross-sectional survey conducted in China with the participants having been in home quarantine for about two months. The focus is on household food-centric matters during the quarantine. First, we describe a new mechanism of household food sourcing that became widely-adopted during the stay-at-home orders, namely community-based online grocery group-ordering (CoGGO). Then, we present differences in food behaviors during the quarantine among different age groups and discuss relevant implications. The findings of the present study can not only help to fill relevant knowledge gaps but also provide insights into future food patterns and policies pertaining to food supply and access, household food security, and food behavior interventions at times of crisis and beyond.

## Materials and methods

### Survey design

The COVID-19 first emerged in China in December 2019. Nationwide stay-at-home orders were in effect by late January and early February, 2020 [20]. In April, an online survey was conducted in China as well as in the United States of America (USA) (stay-at-home directives were issued during mid-March in the USA). The survey requested no identifier items, such as name, address, etc., and involved no medical records or archived samples at all. The study was deemed exempt from requiring human subjects approval by the Institutional Review Board of the University of Pennsylvania. Moreover, potential participants must be 18 years of age or older (self-reported). Once started, they were given the flexibility of skipping any questions or discontinuing at any time.

The survey’s main purpose was to investigate how the COVID-19 pandemic and the stay-at-home directives affected matters relating to food at the household level. The survey included 53 questions (single or multiple responses, numerical or descriptive choices) designed to obtain data on demographics of the participants (e.g., age, number of weeks quarantined at home); food sourcing and vigilance at the market-consumer interface during the quarantine, and parameters reflecting food wastage (food purchasing, at-home food related activities, and food-use behavior), and other relevant household food information such as body weight change (S1 Table).

The questionnaire, initially developed in English, was translated into Chinese. The latter was nearly identical in content to the English version except for a few items that were adapted to be applicable to a Chinese situation, for example, race/ethnicity choices, household income range (in Chinese Yuan instead of USD$), height (in meters instead of feet), and weight (in kilograms instead of pounds). Additionally, the Chinese version contained an extra item in the question of food sourcing once it became evident that the use of the CoGGO food-acquiring mechanism was rapidly spreading throughout China.

### Survey distribution

After pilot testing, both the English and Chinese versions of the survey were placed on the same platform (*Qualtrics*, a web-based survey platform, www.qualtrics.com, United States of America).The distribution of the survey to the public was achieved through individual as well as institutional networks and snowballing via social media. In China, dissemination of the survey was conducted through social media such as QQ and WeChat. In particular, a number of university faculty members participated in the distribution of the survey through a network of former and current students, who scattered around the country while staying-at-home due to the pandemic.

The collection of data online took place from 17 to 27 in April, 2020 (U.S.) and April 22 to May 5, 2020 (China). A comprehensive analysis of the survey data with cross-national comparisons has been published [2]. The present study focuses on two aspects of survey findings that are unique with the Chinese participants that had not been reported elsewhere. First, we identify the community-based online grocery group-ordering as one-of-its-kind food sourcing mechanism that mushroomed in China during the pandemic. Second, we examine how different age groups behaved two-month into the quarantine regarding food attitude and behavior. We discuss broader implications of these findings. It is also necessary to note that the current study is based on a larger pool of Chinese participants (totaling 2126) than that used in the previous report [2] totaling 1732, because of extended online data collection (April 22 to 27 in previous report, April 22 to May 5 in current study).

### Age grouping

Unlike the USA cohort that had a more-or-less even distribution in age groups [2], the Chinese participants were skewed towards a younger demographic. For example, only 2.1% of the 2,126 participants were 60 years of age or older. A recent survey by Zhao et al. investigating dietary diversity in China during the COVID-19 pandemic [21] found a similar phenomenon in which only 16.4% of the participants (n = 1,938) were older than 45 years of age. Despite potential limitations associated with the method of convenience sampling via online survey, meaningful observations can be derived from the given cohort.

The 2,126 participants were divided into three age groups described below. Considerations for the grouping included factors such as psychological maturity, socioeconomic status, and typical food roles based on Chinese culture and family dynamics. The grouping chosen was similar to Bilska et al. [13] whose pre-COVID-19 study investigated the influences of certain demographic factors (age, gender, education etc.) on food waste and food management behaviors among Polish consumers. One exception is that the current study did not separately analyze the responses from 45–59 or 60 and older groups as Bilska et al. Our considerations are discussed below (in Group III).

Group Ⅰ (18–24 years of age, n = 796). Survey participants in this group were most likely to be single, in most cases still attending college or graduate school, and not yet financially independent. The food behavior of this age group was generally affected by greater spontaneity, an alignment towards convenience, and limited experience in food handling or dealing with trade-offs between food consumption priorities and food management [22]. In particular, survey respondents of this age group were most likely staying at home with their parents because the outbreak of the COVID-19 pandemic coincided with the beginning of Chinese New Year, which families traditionally celebrate together. However, their food attitudes and behaviors have a great impact on the family food consumption [23]. For example, the food preferences of these young people while staying at home with families affect the purchasing activities of family food buyers significantly [24], including food category, quantity etc. And these people was easily to waste food more frequently and in larger quantities [25], which has led to a serious food waste in the household level.

Group Ⅱ (25–34 years of age, n = 789). This was a group containing individuals transitioning into the workforce and general society, gradually gaining financial independence, with a growing sense of responsibility.

Group Ⅲ (≥35 years of age, n = 541). In Chinese society, people in their 30s are typically married, with many already young parents. People in this age group were generally financially self-sufficient with a steady income. In particular, these participants would have had an expanding sense of responsibility for their young families, and to a certain extent, their parents. In essence, these people tended to be mature and responsible. Unlike Bilska et al. who had two older groups (45–59 years of age; 60 and older) [13], we did not separate them but included them together in Group III. This was based on two considerations. First, many Chinese families have the tradition of 3-in-1 unity when it comes to daily food matters, with grandparents taking care of food chores (shopping, preparation, cooking) for their busy-working sons or daughters (typically in their 30’s) and grandchildren (typically preschoolers). Our data showed that even the people over 60 are grouped independently, the results were not much difference with the current three age groups, and does not affect the main conclusions of this article (S1 Fig). Second, number of survey respondents older than 45 are small (total 255, or 12% of the cohort). Separating them from Goup III would skew the size of groups, which would undermine the precision and significance of statistical analysis.

### Data analysis

Raw data in *Qualtrics* were exported to SPSS (IBM SPSS Statistics Version 22) for analyses. Descriptive analyses included calculation of means [with 95% confidence interval (95% CI)], standard deviation, median, interquartile range (IQR) of continuous variables and list of categorical variables. Frequency counts and percentages were used for statistical analyses of categorical variables. Chi-square tests were used to compare categorical variables. Analysis of Variance (ANOVA) was used to test continuous variables and for statistical analysis of significance among variables. P<0.05 was set as the standard for statistical significance.

## Results and discussion

### General description

Of the 2,126 respondents, age ranged from 18 to 82 years with a median of 27. A total of 94.4% respondents reported following the stay-at-home orders and having stayed at home for 7–12 weeks (median 10 weeks) at the time of the survey. The remaining 5.6% were presumably in the cadre of emergency or essential personnel with various service or administrative duties. The number of people per household had a median of 4 and a 25-75th percentile of 3 to 5. Table 1 shows a statistical breakdown of the gender, ethnicity, and other aspects of the survey participants.

**Table 1:** Demographic information of survey participants.

Category		Number	Percentage
***Ages***			
	**median, 25%-75% percentile (no. respondents)**	27, 23–35 (N = 2,126)	
	**Ⅰ (18–24)**	796	37.4%
	**Ⅱ (25–34)**	789	37.1%
	**Ⅲ (≥35)**	541	25.4%
***Gender***		N = 1,564	
	**Female**	800	51.2%
	**Male**	713	45.6%
	**Non-binary**	7	0.4%
	**Other**	4	0.3%
	**Prefer not to answer**	40	2.6%
***Race/ethnicity***		N = 1,542	
	**Chinese (Han)**	1,412	91.6%
	**Chinese (Other)**	89	5.8%
	**Prefer not to answer**	41	2.7%
***Total number of people in household* median, 25%-75% percentile (no. respondents)**		4, 3–5 (N = 2,092)	
***Number of people < 18 years in household* median, 25%-75% percentile (no. respondents)**		0, 0–1 (N = 2,057)	
***Household members >65 years of age or otherwise vulnerable*, *for example*, *having chronic heart or lung disease*, *diabetes*, *or immunity disorder*, *Yes***		372	19.3% (N = 1,928)
***Household members tested COVID-19 positive*, *No***		1,922	99.9% (N = 1,924)
***Household members experiencing COVID-19-like symptoms*, *e*.*g*., *dry cough*, *fever*, *tiredness*, *trouble breathing*, *Yes***		37	1.9% (N = 1,928)
***Stayed at home during the pandemic*, *Yes***		1,981	94.4% (N = 2,099)
***Number of weeks staying at home due to the pandemic*? median, 25%-75% percentile (no. respondents)**		10, 7–12 (N = 1,656)	
***Working from home or teleworking currently*? *Yes***		1,037	53.9% (N = 1,925)
***Provincial location of residence according to Zip*** ^***a***^		30	

^a^ According to the postal codes provided by the participants, the provincial-level administrative regions where their families were located were determined. China has a total of 34 provincial administrative regions, including 23 provinces, 5 autonomous regions, 4 municipalities, and 2 special administrative regions.

It is worth noting that the number of responses to individual questions varied, in some cases considerably lower than the grand total (2126) of survey respondents. This is mainly because the survey allowed participants to skip questions or to exit at any time. In particularly, two questions (gender and race) had the lowest responses (26–27% lower than the grand total). This is possibly related to their ‘positioning’, since these two questions were placed in the last part of the questionnaire, making it easy to miss or skip.

### Food sourcing

**Fig 1:**
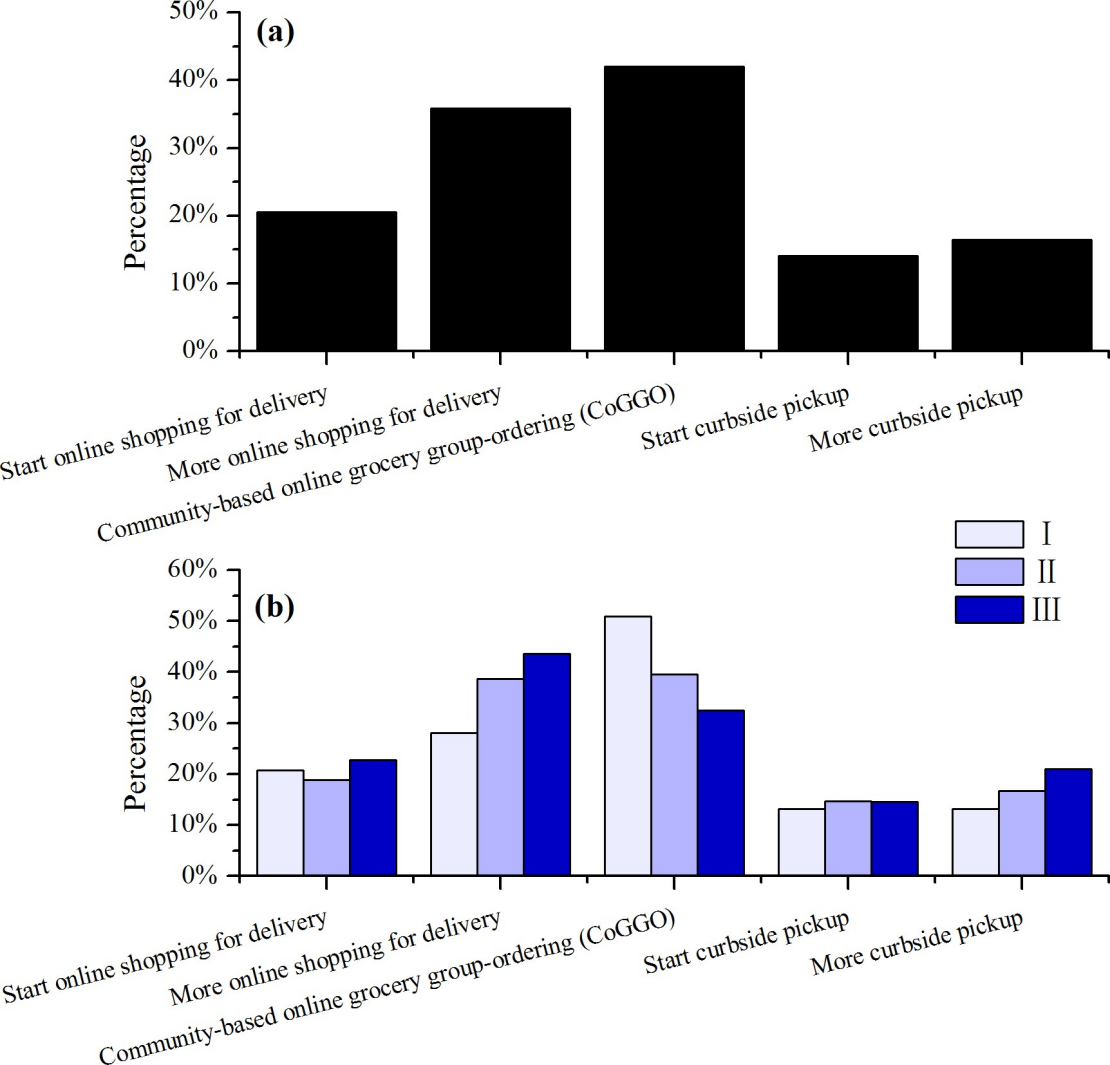
Household food sourcing change during the pandemic compared with before of the whole respondents (a) and different age group (b, p<0.001).

Under the influence of the COVID-19 pandemic, the sources of household food purchases have undergone dramatic changes. There was a sharp increase in online food purchases (Fig 1A). More than half of all respondents reported online grocery shopping for delivery, either for the first time or with a higher frequency (Fig 1A). This shift is not surprising given the unprecedented extent of the health threat and uncertainty posed by the COVID-19 pandemic. Increased online grocery shopping during the pandemic has been reported elsewhere (e.g. [1–5, 26]).

Notably, there were differences in specific online shopping preferences among people of different ages (p<0.001, Fig 1B). Of the respondents in Group III, 66.3% (N = 404) reported purchasing food online for delivery, either for the first time or with increased frequency. In comparison, this food sourcing mode appeared to be proportionally less popular with Group I and II (48.8%, N = 617, and 57.5%, N = 640, respectively). This may be explained that the younger the group, the less responsibility of food shopping in the family: 24.8%, 42.0%, and 56.0% for Groups I, II, and III, respectively (S2 Fig). This also indicated that Group III quickly adopted online food shopping since this group shouldered more responsibility for providing food in households (S2 Fig). People in this group are likely to retain online food purchasing practices in the future, i.e., post-pandemic. Online food purchase here refers to the purchase of food for family consumption through Internet-based e-commerce platforms [27] such as Walmart App, including fresh food, cooked food, etc. A special form of Internet-supported food sourcing during the pandemic, CoGGO, is described later.

On the other hand, the younger age groups showed a stronger preference for community-based online group grocery-ordering (CoGGO, see more details below), with 50.9% (N = 617) of Group I reporting purchasing food especially fresh food via CoGGO compared to 32.4% (N = 404) in Group III. CoGGO is a specific food sourcing format that enables community-based group ordering for all type of foods (fresh or dry, processed or un-processed, plant- or animal-source food) via internet based platform such as WeChat or other Apps. The younger generation has a greater acceptance for novel ideas [6]. They are more adept and adaptable to this new household food sourcing method. It would be of interest to determine whether CoGGO attracted yet greater participation in the later phase of the pandemic and the relevant demographic differentiations.

**Fig 2:**
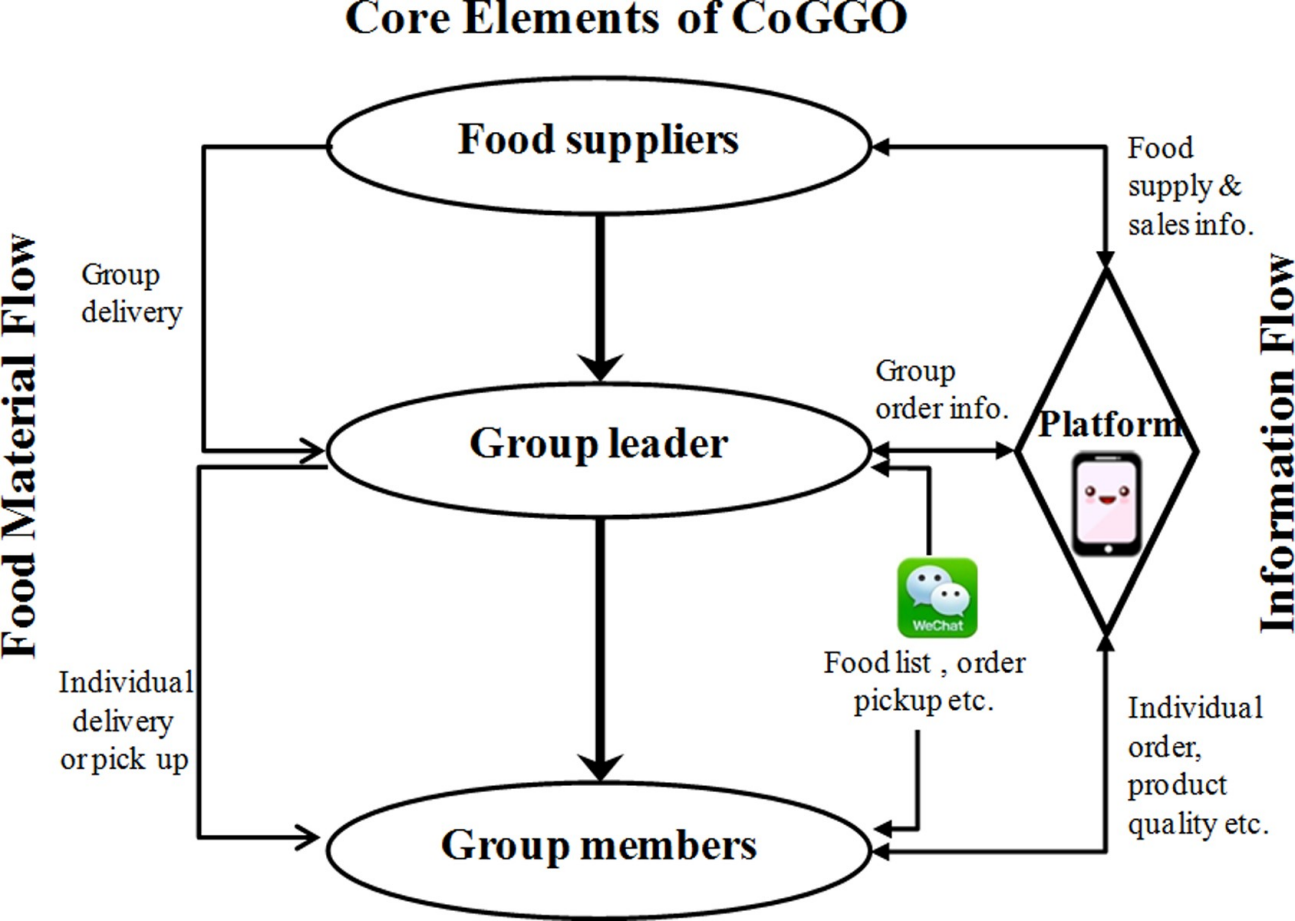
A schematic illustration of CoGGO (community-based online grocery group-ordering) that mushroomed in China during COVID-19 pandemic.

CoGGO as a special form of online-based food acquisition mechanism was reported by 42% (N = 1661) of survey respondents (Fig 1A). To our knowledge, this mechanism of community-based food sourcing for households has not been reported elsewhere. Fig 2 shows the framework and basic function of CoGGO. Under CoGGO, residents in a community or neighborhood form an online group, with a group leader (usually the individual who initiated the community group) acting as the liaison between the group and grocery supplier(s). The digital platform is provided via WeChat, a Chinese mobile phone app that supports the logistics of individualized browsing and selection with a built-in digital wallet to facilitate monetary transaction. For each ‘sale’ event (once or multiple times a week, depending on group size, demands, and food flow), the group leader would share the food inventory with group members, gather orders within a set time frame, and send the order to the supplier for fulfillment, all via the WeChat platform. A group order was usually fulfilled and delivered within a day or two. Delivery was typically to the entrance of the gated community (which is common in Chinese cities) for retrieval by the individuals.

Internet-based group ordering as a form of e-commerce emerged in China prior to the pandemic [27, 28], although pre-pandemic orders tended to mainly be for non-food items or non-perishable foods. The onset of the pandemic quarantine spurred the rapid adoption of CoGGO by households as a new and safer method to obtain food. The adoption of CoGGO apparently spread throughout China as survey participants represented 30 out of 34 provincial-level administrative regions. Interestingly, a recent report from an industry-sponsored survey provided additional evidence of the rapid growth of CoGGO in China, with fruits, vegetables, meats, and eggs topping online food sales (https://www.iimedia.cn/c400/74181.html).

The potential implications of CoGGO as an alternative mechanism for household food sourcing during the pandemic are multifaceted. Grocery stores in Chinese cities are oftentimes tightly spaced and overly crowded. CoGGO provided a safer alternative approach for households to meet their food requirements while cutting down potential viral exposure, thereby helping to keep viral infections down. Furthermore, CoGGO events helped to cultivate a sense of community connection among participating members (oftentimes neighbors) at a time of physical isolation and psychological stress. The “buying group” model described by Si et al. as organized by residents in Wuhan had a similar effect [6]. Further research is needed to address several issues, such as how CoGGO has evolved post-pandemic, how it may affect food distribution efficiencies as well as potential carbon footprints, what long-term impacts it may have pertaining to food system resilience, food access and security at the household level, as well as food safety and food justice.

### Food vigilance

**Fig 3:**
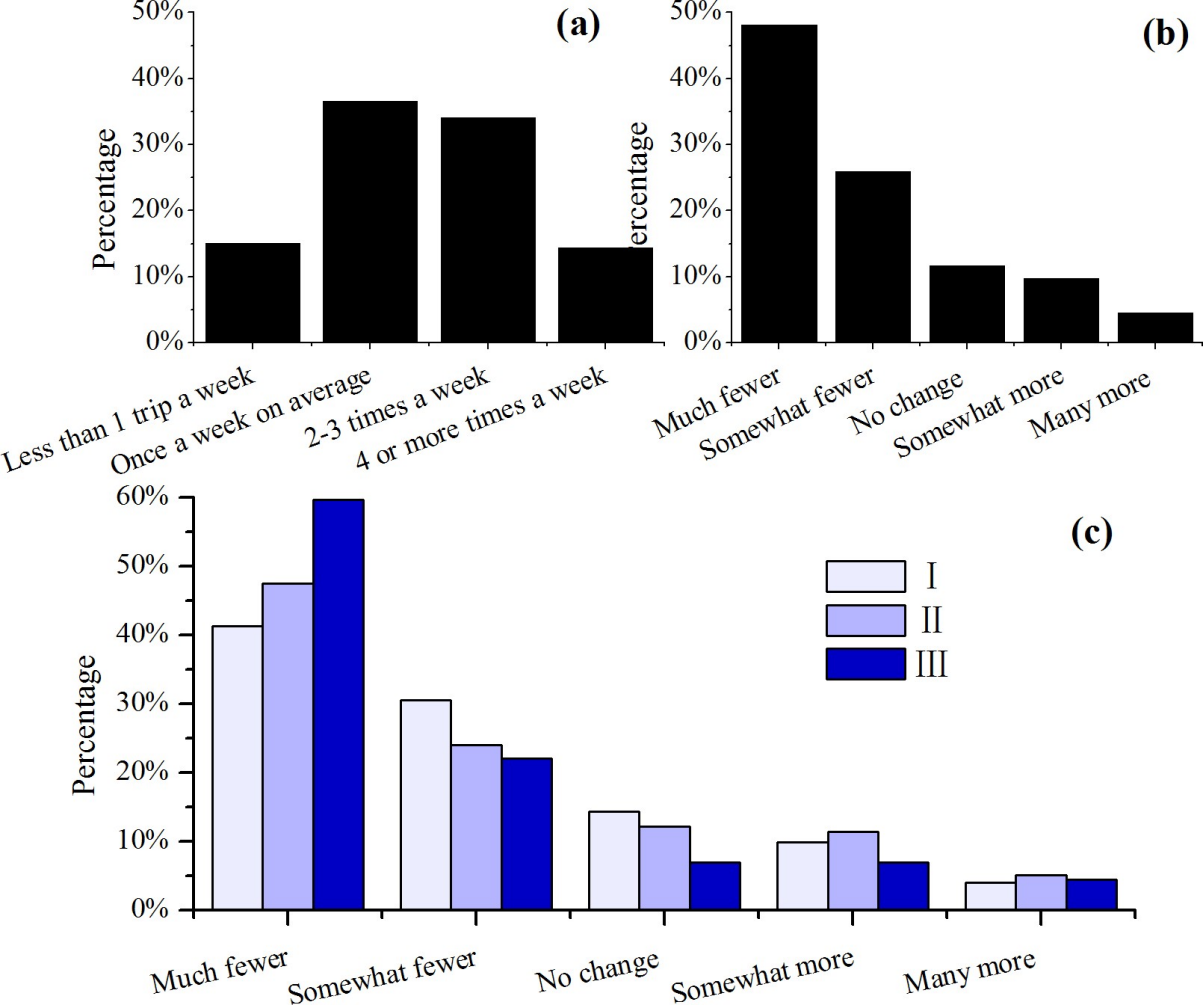
Frequency of grocery shopping trips before the pandemic (a), changes during the home quarantine (b), and age groups differed in cutting down grocery shopping trips (c, p<0.001) during the COVID-19 pandemic.

36.5% of the respondents reported that the frequency of food shopping trips prior to the pandemic was once a week on average, 34.4% of them was 2–3 times a week, 14.4% of them was 4 or more times a week, and 15.0% reporting a frequency of less than weekly (Fig 3A, N = 1749). However, survey participants reported far fewer food shopping trips to marketplaces during the pandemic (Fig 3B). Participants compensated for the reduced number of physical trips to the grocery store by increasing online-based food acquisition (Fig 1B).

In terms of age difference, there was no difference in the frequency of food shopping prior to the pandemic (S3 Fig). In contrast, during the home quarantine, a higher proportion (59.6%, N = 431) of respondents in Group III reported “much fewer” grocery shopping trips compared to Groups I and II of 41.3% (N = 651) and 47.5% (N = 668) lower, respectively (Fig 3C). Those reporting “much fewer” plus those reporting “somewhat fewer” grocery trips accounted for 81.7% (N = 431) in Group III, compared to 71%–72% in the younger groups. This result can be attributed to the older age group exhibiting a higher propensity to protect themselves and families from potential viral-exposure risks.

Participants also compensated for reducing physical trips to grocery stores by purchasing extra food compared to before the pandemic (Table 2). The five food categories in which this pattern was most prevalent were vegetables, meat/fish/eggs, rice/flour/dried beans, fruits, and milk and dairy, and this pattern was consistent across all age groups. Not surprisingly, 46.6%–56.4% of respondents in Group III reported purchasing extra food within these five food categories, whereas substantially lower percentages were reported in Group I (29.9%–44.0%), and that in Group II falling between the two extremes. This result is an additional indication that Group III demonstrated a heightened sense of responsibility for household food security, consistent with Chinese culture and family structure. It should be noted that the particular question in the questionnaire relating to extra food purchases was descriptive/qualitative rather than quantitative. Therefore, it could not be determined whether any of the reported extra purchases may have constituted stockpiling or hoarding. It is assumed that this was not the case since the Chinese respondents gave a higher rating to food supply availability and diversity at the market-consumer interface compared to the respondents in the USA survey [2].

**Table 2:** Percentage of survey participants reporting extra food purchases during the COVID-19 home quarantine compared to before the pandemic^a^.

Food Category	Group			P ^b^
	Ⅰ	Ⅱ	Ⅲ	
Meats/fish/eggs	N = 602	N = 637	N = 417	< 0.001
	34.9%	43.3%	52.5%	
Milk & dairy	N = 592	N = 628	N = 401	< 0.001
	29.9%	36.3%	46.6%	
Fruits	N = 589	N = 631	N = 407	0.002
	37.4%	43.6%	48.4%	
Vegetables	N = 593	N = 633	N = 413	< 0.001
	44.0%	52.1%	56.4%	
Manufactured grain products	N = 587	N = 625	N = 404	0.004
	27.3%	31.2%	37.1%	
Rice/flour/dried beans	N = 589	N = 631	N = 407	< 0.001
	38.9%	44.4%	51.6%	
Frozen food	N = 583	N = 621	N = 393	0.002
	25.7%	31.7%	35.4%	
Canned/jarred food	N = 583	N = 618	N = 381	0.001
	16.1%	18.9%	21.3%	
Snacks/sweets	N = 588	N = 620	N = 393	0.045
	23.8%	26.8%	28.5%	

^a^ "extra food purchases" refers to general purchasing patterns of various food types as compared to before the pandemic.

^b^ p-value < 0.05 indicates statistical significance using ANOVA method.

### Food waste reduction

**Fig 4:**
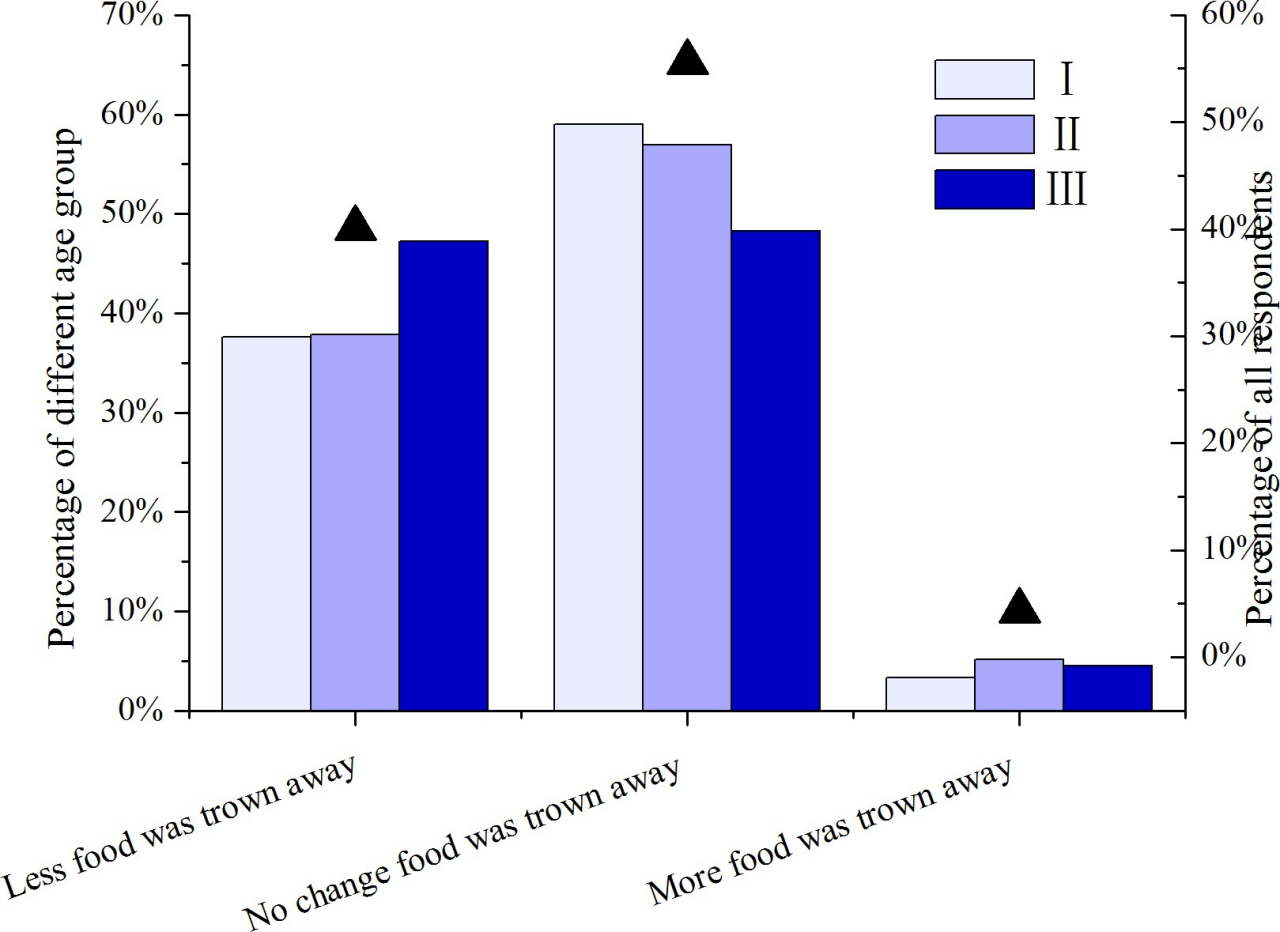
Wasted food amount change from all respondents (Solid triangle) and different age groups (bar) during the pandemic (p = 0.006).

Roughly 40.1% (N = 1612) of all respondents reported less food wastage during the home quarantine compared to prior to the pandemic (Fig 4). A greater proportion (47.2%, N = 398) of respondents in Group III reported less food wastage than the other two groups (37.6%, N = 598 and37.8%, N = 616) (Fig 4). This result was consistent with that of several pre-pandemic studies [9–13]. The three reasons given most often in the current study among all age groups for wasting less food were: (1) a greater appreciation of food and awareness of a potential shortage; (2) more time for meal planning and cooking; (3) avoidance of needing to visit the grocery store (Table 3). Respondents in Group I (53.5%, N = 591) indicated that they had more time for meal planning and cooking, thereby leading to less food waste. Within Group III, respondents gave the avoidance of needing to visit the store as the most important reason for reducing food wastage (Table 3). There were significant differences in attitudes to food between the different age groups (p = 0.037). Although motivations for reducing food waste reduction may have diminished as the home quarantine was lifted in China, it remains unclear whether this resulted in a return to pre-pandemic food waste levels. Nevertheless, most of GroupⅠrespondents (51.2%, N = 598) indicated an awareness of food wastage issues, and nearly one-third (32.8%, N = 591) reported being more creative in using food items during the lockdown. It is possible that the positive impact of the pandemic on food behavior (e.g., wasting less food) may persist beyond the duration of the lockdown. It is important to develop effective interventions to extend the positive changes that took place during the pandemic and to engage the public to encourage sustainable food behaviors.

**Table 3:** Reasons, categories and behaviors for discarding food during COVID-19 quarantine, as reported by different age groups.

		Ⅰ	Ⅱ	Ⅲ	P a
Reasons for wasting less		N = 591	N = 613	N = 401	0.037
	More appreciative of food, more aware of potential food shortage	61.6%	53.8%	53.1%	
	More time for meal planning and cooking	53.5%	48.3%	51.6%	
	Tried to make more to avoid going to the stores	52.1%	51.2%	52.6%	
	Became more creative in using food items	32.8%	28.9%	33.7%	
	Less sensitive to expiration dates	11.8%	10.1%	12.5%	
**Type of foods thrown away recently**		N = 589	N = 609	N = 399	0.066
	Spoiled fruits or vegetables	61.0%	61.9%	60.7%	
	Leftovers from home cooking	45.0%	42.2%	39.6%	
	Food past expiration date	42.1%	37.1%	39.1%	
	Food left on someone’s plate	30.4%	26.3%	36.3%	
	Leftovers from restaurant take-out	20.9%	24.3%	14.5%	
	Unfinished canned or jarred or packaged food items	17.3%	16.7%	13.3%	
	Stale bread or bakery goods	15.4%	18.1%	16.0%	
	Uncooked or un-used food no longer wanted	7.5%	9.9%	8.0%	
**Actions prior to keep-or-toss decision**		N = 584	N = 609	N = 423	
Check expiration dates		75.2%	73.7%	59.1%	<0.001
Smell if it’s still okay		67.1%	63.7%	50.8%	0.001
Peel or cut off bad or moldy portion and use the remaining		49.1%	48.1%	38.8%	0.027
Try to create new dish using leftovers		44.3%	39.6%	33.3%	0.039

^a^ p-value < 0.05 indicates statistical significance using ANOVA method.

The responses of participants to a question relating to the types of food thrown away prior to the survey (i.e., as remembered most accurately) were most often spoiled fruits and vegetables, followed by leftovers of home cooking, and finally food past the expiration date (Table 3). Interestingly, most respondents (59.1%–75.2%) reported checking the expiration date of food items before deciding on whether to discard them, suggesting the critical importance of date-labeling in influencing consumer food waste behavior, even during times of crisis. Respondents also reported using sensory-based judgments (e.g., smelling if the food is still okay) to decide whether to dispose of food items. Furthermore, respondents commonly reported cutting off spoiled parts of fresh fruit and vegetables or creating a new dish from leftover food (Table 3).

Positive behavioral changes including reductions in food wastage during the COVID-19 pandemic have been reported more broadly. For instance, Jribi et al. found that most of the respondents in a small-scale survey (n = 284) in Tunisia reported having food conservation strategies in place, with nothing of what they bought being discarded [18]. In an Italian survey [29] found that most of the surveyed 1,078 households reported a reduction in food waste during the pandemic lockdown. Positive food attitudes with more prudent use of food and less wastage were attributed to heightened awareness and uncertainties people were facing regarding the trajectory of COVID-19 [2]. Regardless of the pandemic, food waste is a serious challenge undermining global food security and sustainability [30], and therefore reducing food loss and waste throughout the food chain, particularly at the consumer level, is paramount [31]. In addition, finding ways to sustain the positive changes and to broaden relevant reach is vitally important within the pursuit of a resilient and sustainable food future.

### Weight change

**Fig 5:**
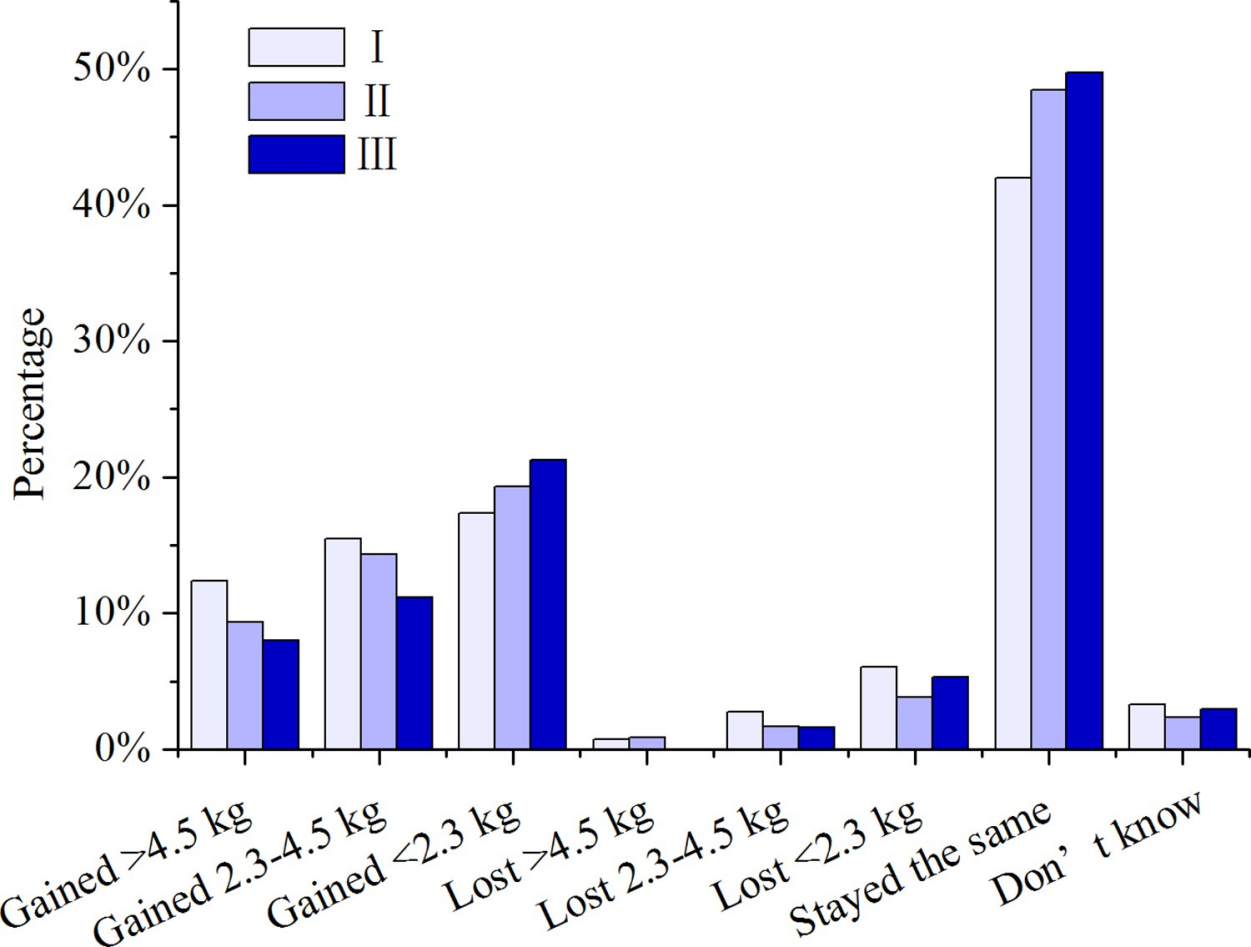
Body weight change during the COVID-19 pandemic in different age groups (p = 0.018).

After being housebound for about two months with limited physical space and restricted daily activities, self-reported weight-change differed between age groups (Fig 5). Notably, those reporting weight gain of >4.5 kg (clinically significant weight gain) accounted for 12.4% (N = 581) in Group I, compared to 9.3% (N = 601) and 8.0 (N = 376) in Groups II and III, respectively. Respondents reporting >2.3 kg weight gain (i.e. the two highest categories added together) accounted for 27.9% in Group Ⅰ, 23.6% in Group Ⅱ, and 19.1% in Group Ⅲ. The greatest proportion of all age groups reported no weight change (42.0%, 48.4%, and 49.7% for Group I, II, and III, respectively). Only a small percentage in each group indicating weight loss, mostly in the <2.3 kg category (Fig 5).

There have also been reports of pandemic-induced changes to weight more broadly. For example, Ammar et al. in a survey of 1,047 participants in Africa, Asia, and Europe identified that home quarantine negatively affected food consumption patterns, with overeating was reported [19]. In a survey conducted in Italy, 19.5% of participants reported gaining weight and 42% indicated greater intake of comfort food (chocolate, dessert, ice cream, etc.), which was attributed to higher anxiety [17]. In our previous report comparing U.S. vs. Chinese cohorts [2], the Chinese cohort (n = 1,732) had 11% self-reporting >4.5 kg weight gain, compared to just ~2% of the USA cohort (n = 1,547). Weight change may be related to alteration in food purchasing and eating patterns etc. [2, 17], which could reflect the impact caused by the household food consumption behavior changes during the pandemic to some extent. Apparently, the younger Chinese participants were more prone to weight gain under the same conditions of home quarantine as their older counterparts. This may be explained as the young people might have enjoyed delicious home-made meals while staying with parents during the quarantine. They may also be more resilient to stress or life-changing events under the given circumstances in the extend-family structure in China. This is mainly because they are not financially independent, therefore their senses of financial responsibilities and worries are yet to fully develop. The implications of such resilience as well as the susceptibility to weight-gain and long-term health implications, if any, would be of interest for future research.

### Other food consumption behaviors

Additionally, the age groups differed significantly on the following issues: younger people were more optimistic pertaining to perceived food security and food supply stability locally or nationally; the older ones indicated more frequent home-cooked meals as well as household members cooking/eating together more often; the older group were also more in charge for household food matters such as food handling and cooking (S2, S4 and S5 Figs). On the other hand, there were no differences between age groups regarding: (1) changes (increase or decrease) in types of food consumed during vs. before the pandemic, which were consistent across the three groups; (2) most respondents regardless of age group expressed that the COVID-19 pandemic would likely influence their food behavior in the future concerning food purchase or food handling; (3) food waste disposal was primarily via garbage services, alternatives such as composting was minor.

Considered together, the results of this survey indicated that people adjusted and changed their food consumption behaviors while responding to the impact of the pandemic, with these changes differing among different age groups. Understanding these changes and following up on the impact of the pandemic is important to enhance the resilience of the food system to shock events. Key points of knowledge and implications can be derived from the survey results, including: (1) The pandemic-related lockdown accelerated the development of online food shopping, and particularly in the use of CoGGO as a new household food-sourcing for households. Going forward, the regulation of the new online food supply methods and the balance between online and traditional food supply modes will present a challenge to policy makers and business leaders. (2) In this study, under the dual pressure of an increased demand for food at home and a substantial reduction in the availability of in-store food during the pandemic, Group III respondents rapidly adopted online-based food sourcing methods, including CoGGO. Future development of food supply channels/mechanisms to target the group of consumers shouldering most of household food-purchasing responsibilities, i.e., the older group, will be important to ensure household food security under a pandemic crisis. (3) retaining changes in food disposal behavior among the different age groups that occurred due to the influence of the pandemic through to the post-pandemic period can help to reduce food wastage in the future. In particular, it is important to retain changes seen in younger people of becoming more aware of potential food shortages and the uptake of improved food processing methods. On the other hand, further study from a public health perspective on gains in weight among younger people under the extended family structure in China may be warranted.

## Conclusions

The present study used convenience-snowball sampling and real-time data collection through an Internet-based survey to obtain data on the food experience of people in China during the COVID-19 pandemic-related quarantining. The pandemic changed how people sourced and waste food. However, these changes were significantly different among different age groups. The group with more family responsibilities (i.e., Group III) demonstrated heightened vigilance and maturity in managing food matters at a time of extraordinary challenges and uncertainty. The patterns of food behavioral change exhibited by the different age groups were consistent in direction (increase or decrease) and ranking order (e.g., Group I > Group II > Group III, or *vice versa*). The observed changes and adaptations reflected the resilience and ability of the respondents to deal with household food matters at times of extreme challenge. These differences based on age group in household food consumption behaviors have a decisive role in policy making such as food supply methods development, food intake and nutrition guidelines for young people, optimization of online food sourcing methods etc. For example, foods such as vegetables and fish that older people prefer to buy could be placed in a more prominent position on the online shopping webpage, making it easier to find. The rapid spread and wide adoption of CoGGO is of interest for further research pertaining to matters of food supply, food safety, and public health, as well as the rippling effects on socioeconomic and sustainability issues.

The changes in food behavior demonstrated in the current study are highly contextual in that they were directly related to or triggered by the COVID-19 pandemic and the need to quarantine at home. Future studies should focus on lessons that can be learned to enhance preparedness in dealing with future crises, identifying findings that may be relevant and applicable for future times of normalcy, identifying which behaviors can be sustained with lasting impact and which may fade away once the viral threat subsides, and what actions or mitigation strategies should be taken to sustain and counter the positive and negative changes, respectively. Moreover, independent analysis of older age group (45–59 years of age and 60 above) needs further scrutiny, since they seem to be more resilient to psychological pressure during the pandemic [2].

Food is a basic necessity of mankind. The results of the current study offer insight into the resilience of the population and the food system in respect of food choices, innovations in food-sourcing, flexibility in food handling, and adaptiveness during the COVID-19 crisis. The results of the present study can also reveal required research into future food patterns and show changes to food behavior at times of crisis and the relevant consequences.

One of the limitations of the current study was that convenience-based sampling is not representative, and therefore the findings are not generalizable [32, 33]. However, the classic issue of sample representativeness may not be as critical in the present situation considering the widespread impacts of the pandemic, essentially encompassing everyone in society [2]. It is also necessary to note that although the present study focused on examining behavioral changes of different age groups during the COVID-19 quarantine, various factors can influence consumer food attitudes and behavior, e.g., gender, education, socioeconomic status, and personal experience. The complexity of the interrelated factors and their impact on food behaviors deserve further research.

## Supporting information

S1 Fig Purchasing household food roles of different age groups based on two different age grouping method. (a) Three age groups with grouping ≥35 years old; (b) four age groups with splitting the older group of 45–59 and 60 above separately (p<0.001).
(TIF)

S2 Fig Different age groups in household food roles (p<0.001). (a) Grocery shopping; (b) Meal planning and cooking. It showed the older group were more in charge for household food matters such as food handling and cooking.
(TIF)

S3 Fig Frequency of grocery shopping trips before the pandemic by different age groups. It showed frequency of food shopping trips did not differ between the age groups before the pandemic with p = 0.067.
(TIF)

S4 Fig Perception of food security before (a, p = 0.003) and during (b, p<0.001) the COVID-19 pandemic. It indicated the younger people were more optimistic pertaining to perceived food security and food supply stability locally or nationally.
(TIF)

S5 Fig Household cooking situations during the pandemic (p<0.001). (a) home meals cooking, (b) home members cooking/eating together. It showed the older ones indicated more frequent home-cooked meals and cooking/eating together more often.
(TIF)

S1 Table Main questions of the survey. (PDF)
