# Supplementary figures and images for: COVID-19 affected the food behavior of different age groups in Chinese households
Source: PLoS One. 2021 Dec 17;16(12):e0260244. doi: 10.1371/journal.pone.0260244 (PMC8682873; doi:10.1371/journal.pone.0260244)

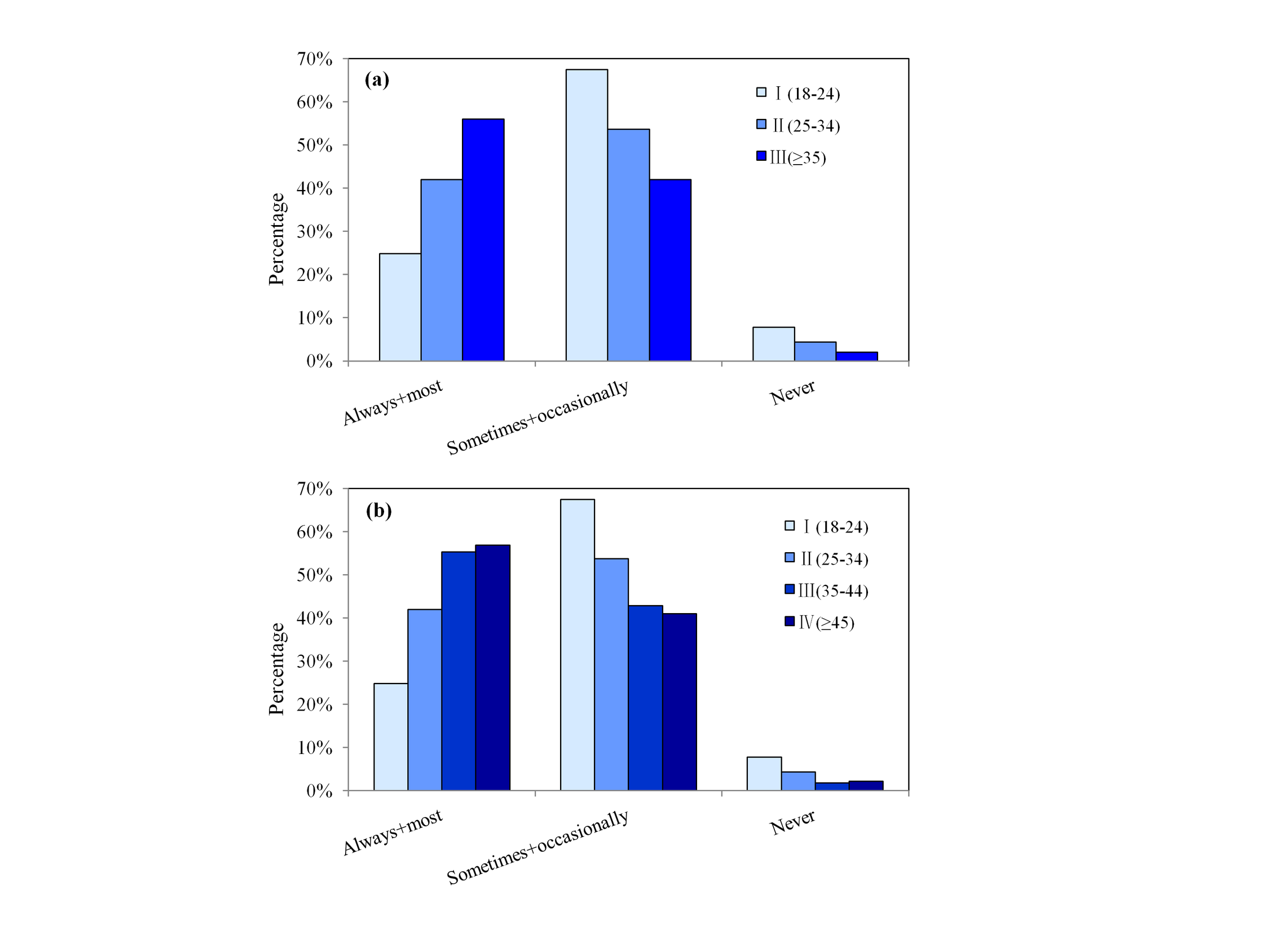

Supplement: S1 Fig — (a) Three age groups with grouping ≥35 years old; (b) four age groups with splitting the older group of 45–59 and 60 above separately (p<0.001). (TIF) [file pone.0260244.s001.tif]

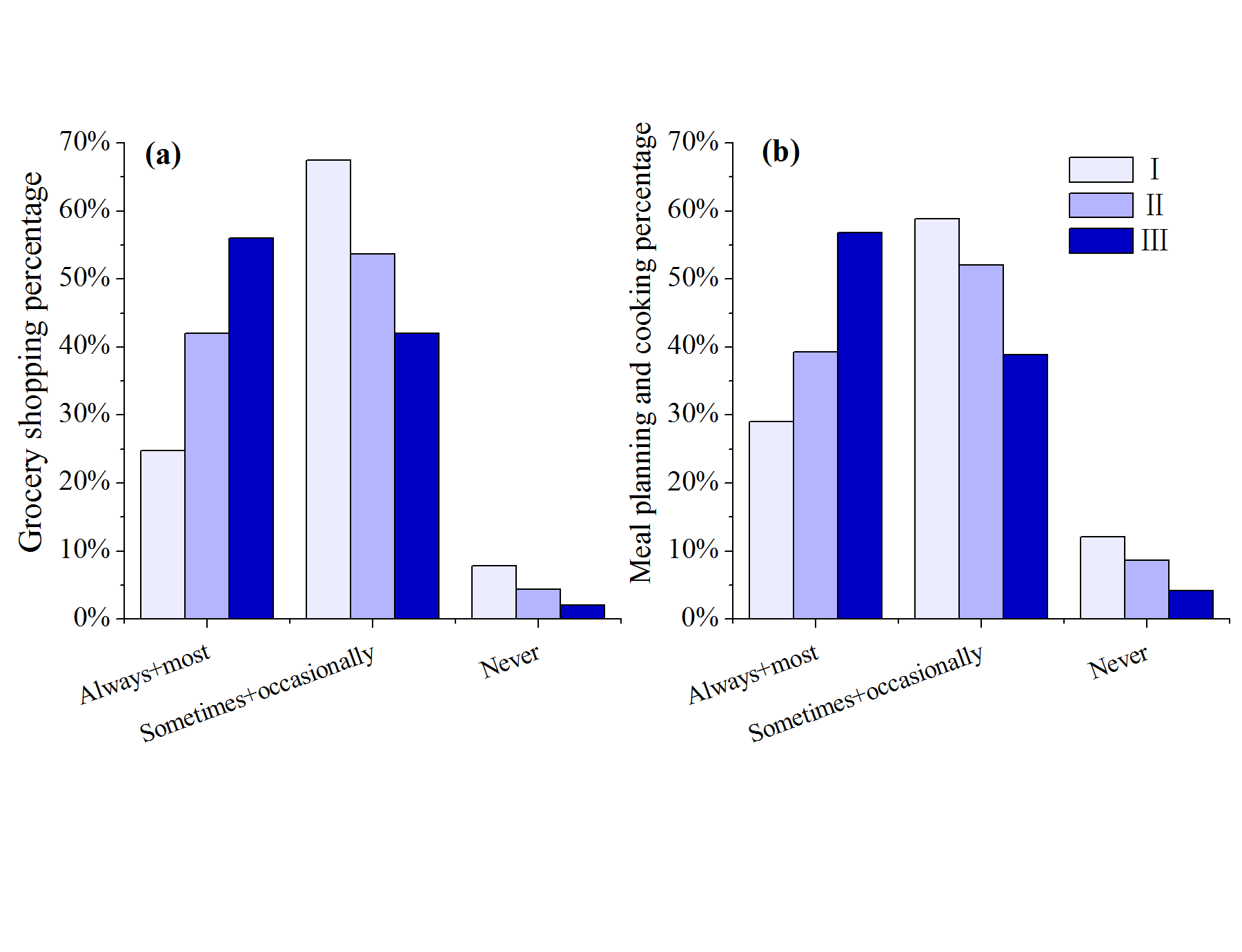

Supplement: S2 Fig — (a) Grocery shopping; (b) Meal planning and cooking. It showed the older group were more in charge for household food matters such as food handling and cooking. (TIF) [file pone.0260244.s002.tif]

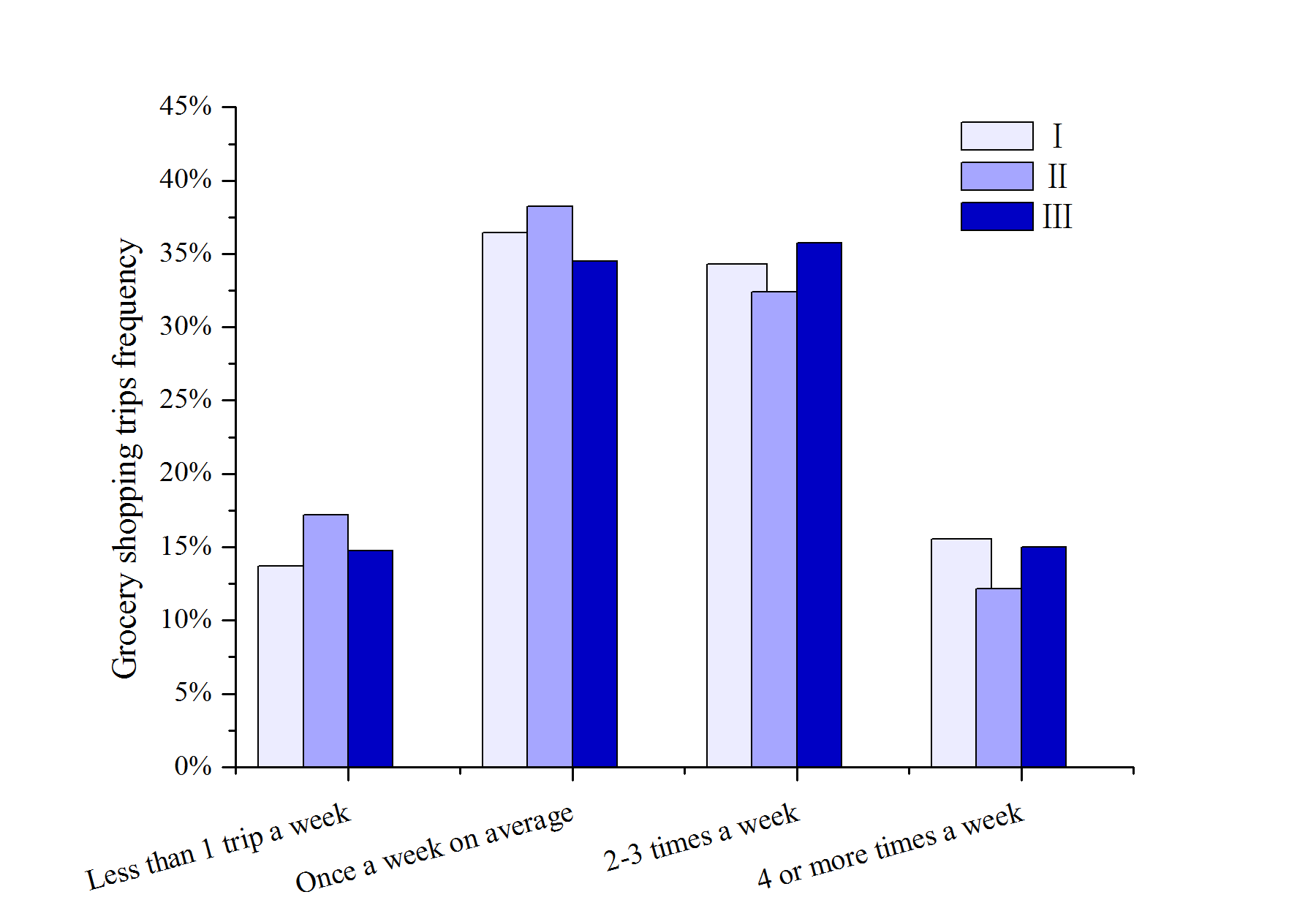

Supplement: S3 Fig — It showed frequency of food shopping trips did not differ between the age groups before the pandemic with p = 0.067. (TIF) [file pone.0260244.s003.tif]

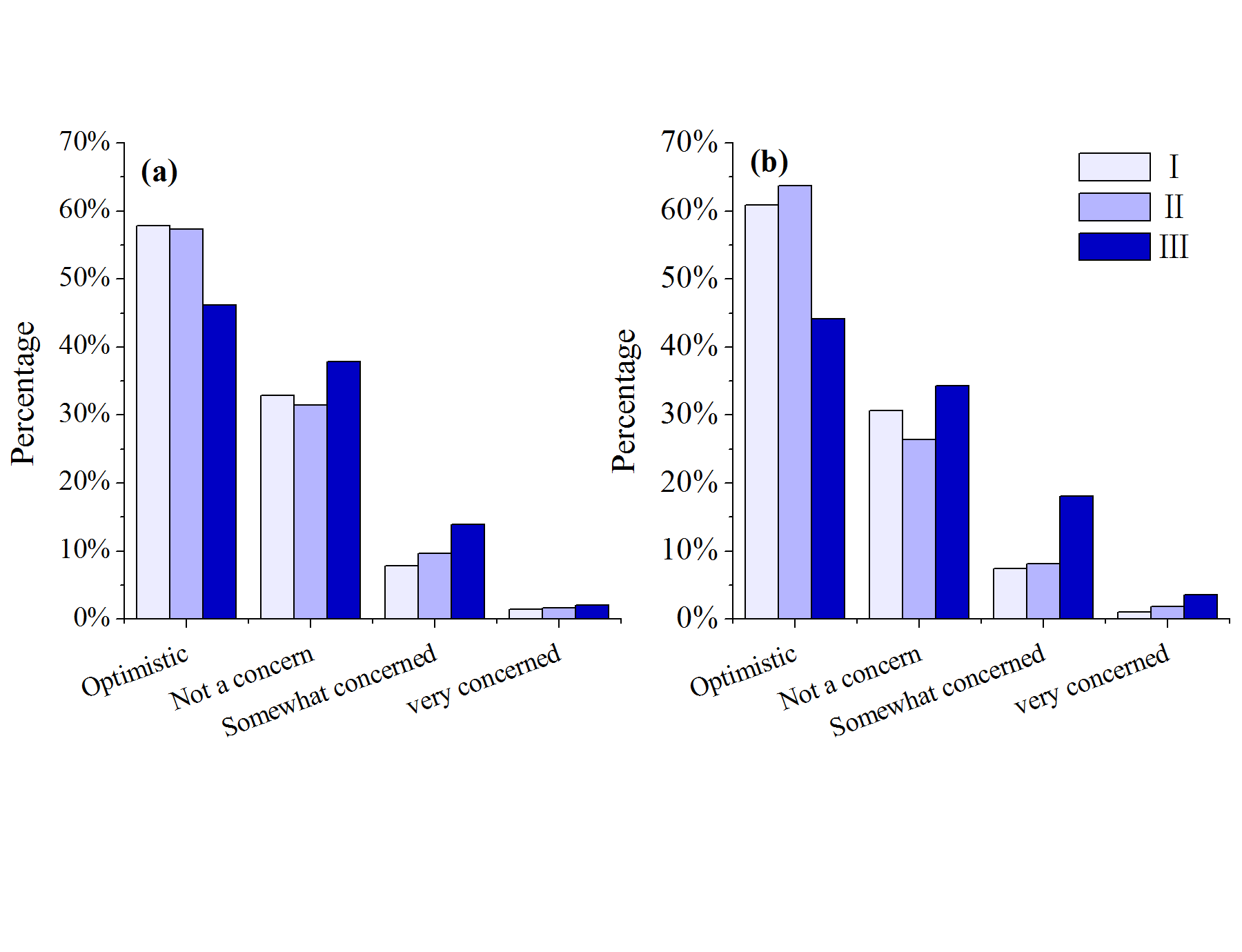

Supplement: S4 Fig — It indicated the younger people were more optimistic pertaining to perceived food security and food supply stability locally or nationally. (TIF) [file pone.0260244.s004.tif]

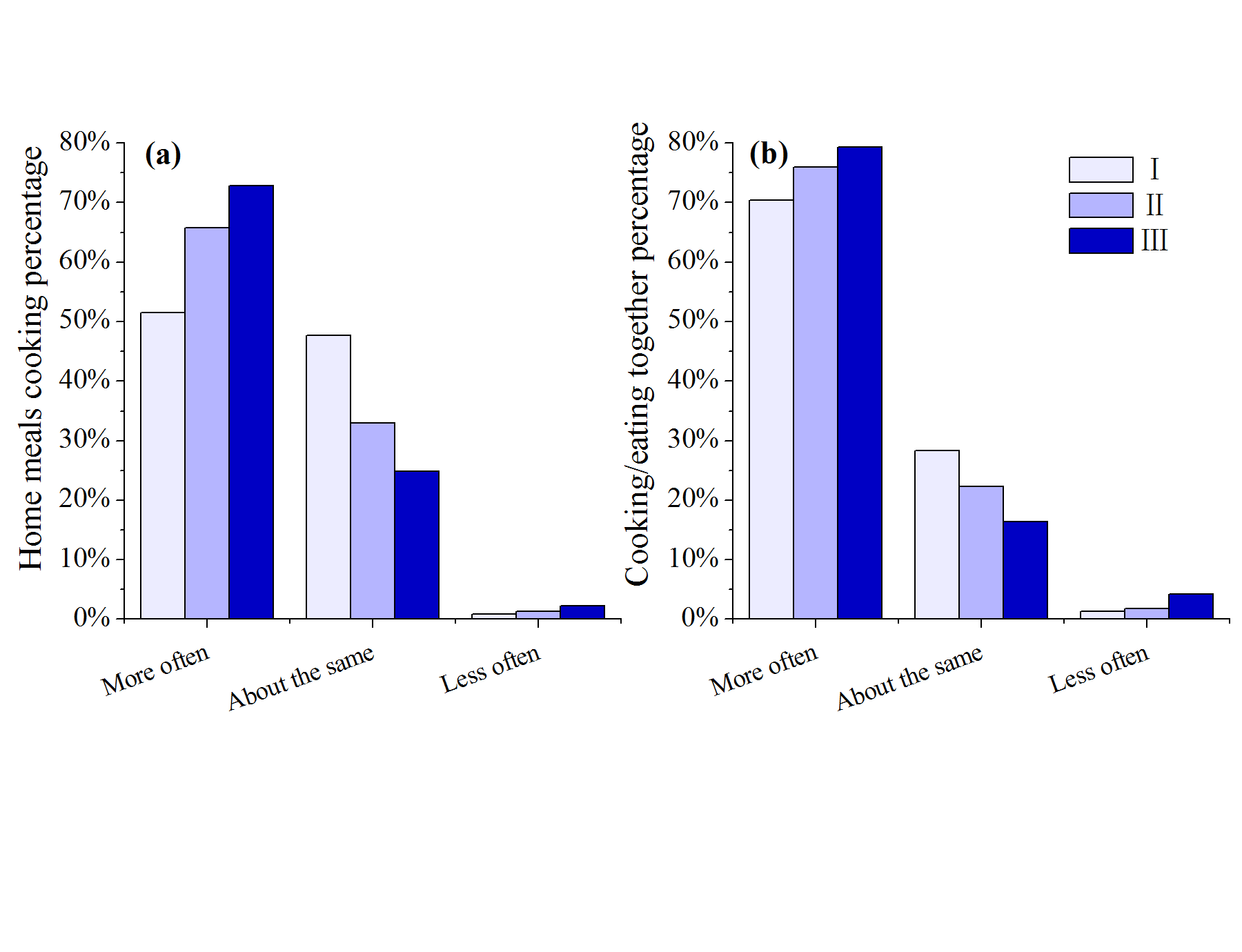

Supplement: S5 Fig — (a) home meals cooking, (b) home members cooking/eating together. It showed the older ones indicated more frequent home-cooked meals and cooking/eating together more often. (TIF) [file pone.0260244.s005.tif]
