# Supplementary material for: COVID-19 affected the food behavior of different age groups in Chinese households
Source: PLoS One. 2021 Dec 17;16(12):e0260244. doi: 10.1371/journal.pone.0260244 (PMC8682873; doi:10.1371/journal.pone.0260244)
Supplement: S1 Table — (PDF) [file pone.0260244.s006.pdf]

S1 Table. Main questions of the survey

Ⅰ. Food Consumption and Waste Behaviors

Food Sourcing During The Pandemic -Restaurant food ordering

1. Compared to before the pandemic, did you order restaurant food:

Much more often; Somewhat more often; No change; Somewhat less often; Much less often

2. Compared to before the pandemic, how much have you spent on restaurant food take-out orders:

Spent much more money; Spent somewhat more money; No change;
Spent somewhat less money; Spent much less money

Food Sourcing During The Pandemic -Grocery shopping:

3. Before the pandemic, how often did you or your household make trips to stores/markets to buy groceries?

Less than 1 trip a week; Once a week on average; 2-3 times a week; 4 or more times a week

4. During the pandemic, has your household made more or fewer trips to buy groceries:

Many more trips; Somewhat more trips; No change; Somewhat fewer trips; Much fewer trips

5. During the pandemic compared with before, did you: (select all that apply)

For the first time, start ordering groceries online to be delivered;
Order more groceries online to be delivered;
For the first time, start ordering groceries online to be picked up outside the store;
Order more groceries online to be picked up outside the store;
More community-based online group grocery-ordering for delivered

Food consumption

6. During the pandemic when shopping, did you buy extra (or less) amounts compared to your normal routine?

	Bought extra	No change	Bought less
Meats/fish/eggs			
Milk & dairy			
Fruits			
Vegetables			
Manufactured grain products, e.g. bread/pasta			
Rice/flour/dried beans			
Frozen food			
Canned/jarred food			
Snacks/sweets			
Bottled water			
Alcoholic beverages			

7. During the pandemic as compared to before, are you or your household eating more of certain types of food but less of other types:

	Much more	Somewhat more	No change	Somewhat less	Much less
Meats/fish/eggs					
Milk & dairy					
Fruits					
Vegetables					
Manufactured grain products					
Rice/flour/dried beans					
Frozen food					
Canned/jarred food					
Snacks/sweets					
Bottled water					
Alcoholic beverages					

8. During the pandemic, did you try to be more prudent/frugal about meal planning or spend less money on meals? Y/N
9. During the pandemic, have you made a second meal or snack of leftovers from restaurant food? Y/N

Food wastage

10. Did you know that people in mid- to high-income countries generally throw away one fifth of their food? Y/N
11. If the average person throws away one fifth of their food, before the pandemic about how much food did your household throw away?
    Much more Somewhat more About 1/5 Somewhat less Much less
12. During the pandemic compared to before, did your household throw away:
    More food No change Less food
13. Please select all of the reasons that you may have thrown out less food
    More appreciative of food, and more aware of potential food shortage;
    More time for meal planning and cooking;
    Less sensitive to expiration dates;
    Tried to make more out of what we have to avoid going to the stores;
    Became more creative in using food items
14. Recently (in the past week or days as you can recall), what foods did your household thrown away (select all that apply):
    Spoiled fruits or vegetables;
    Old sauces or un-finished canned or jarred or packaged food items;
    Stale bread or bakery goods;
    Leftovers from restaurant take-out;
    Leftovers of home cooking
    Food left on someone's plate
    Uncooked or un-used food no longer wanted (bought too much)
    Food past expiration date
15. When deciding whether or not to throw away food, do you: (select all that apply)

	Before pandemic	During pandemic
Check expiration dates		
Smell if it's still okay		
Peel or cut off bad or moldy portion and use the remaining		
Try to create new dish using leftovers, for example, making stew or chef's pie		

16. What do you do with the food that is thrown away? Select all that apply:

	Before pandemic	During pandemic
Trash bin or garbage collection services		
Down the drain (in-sink garbage disposal)		
Backyard composting		
Community composting		
Feed to pets		
Feed to farm animals		
Left to wildlife		
Other		

Ⅱ. Impact of the Pandemic on Food Consumption in the Future

17. Do you think the pandemic may change the type of foods you will purchase in the future?
    Most likely Somewhat likely Unlikely Not sure
18. Do you think the pandemic may change how you will treat/handle food in the future?
    Most likely Somewhat likely Unlikely Not sure
